# Lack of Detection of Bt Sugarcane Cry1Ab and NptII DNA and Proteins in Sugarcane Processing Products Including Raw Sugar

**DOI:** 10.3389/fbioe.2018.00024

**Published:** 2018-03-27

**Authors:** Adriana Cheavegatti-Gianotto, Agustina Gentile, Danielle Angeloni Oldemburgo, Graciela do Amaral Merheb, Maria Lorena Sereno, Ron Peter Lirette, Thais Helena Silva Ferreira, Wladecir Salles de Oliveira

**Affiliations:** ^1^Regulatory Department, Centro de Tecnologia Canavieira (CTC), Piracicaba, Brazil; ^2^Biotechnology Department, Centro de Tecnologia Canavieira (CTC), Piracicaba, Brazil; ^3^Ron Lirette Biotech Consulting LLC, Theriot, LA, United States

**Keywords:** sugar, highly purified substance, sugarcane, Cry1Ab, neomycin-phosphotransferase type II

## Abstract

Brazil is the largest sugarcane producer and the main sugar exporter in the world. The industrial processes applied by Brazilian mills are very efficient in producing highly purified sugar and ethanol. Literature presents evidence of lack of DNA/protein in these products, regardless of the nature of sugarcane used as raw material. Recently CTNBio, the Brazilian biosafety authority, has approved the first biotechnology-derived sugarcane variety for cultivation, event CTC175-A, which expresses the Cry1Ab protein to control the sugarcane borer (*Diatraea saccharalis*). The event also expresses neomycin-phosphotransferase type II (NptII) protein used as selectable marker during the transformation process. Because of the high purity of sugar and ethanol produced from genetically modified sugarcane, these end-products should potentially be classified as “pure substances, chemically defined,” by Brazilian Biosafety Law No. 11.105. If this classification is to be adopted, these substances are not considered as “GMO derivatives” and fall out of the scope of Law No. 11.105. In order to assess sugar composition and quality, we evaluate Cry1Ab and NptII expression in several sugarcane tissues and in several fractions from laboratory-scale processing of event CTC175-A for the presence of these heterologous proteins as well as for the presence of traces of recombinant DNA. The results of these studies show that CTC175-A presents high expression of Cry1Ab in leaves and barely detectable expression of heterologous proteins in stalks. We also evaluated the presence of ribulose-1,5-bisphosphate carboxylase/oxygenase protein and DNA in the fractions of the industrial processing of conventional Brazilian sugarcane cultivars. Results from both laboratory and industrial processing were concordant, demonstrating that DNA and protein are not detected in the clarified juice and downstream processed fractions, including ethanol and raw sugar, indicating that protein and DNA are removed and/or degraded during processing. In conclusion, the processing of conventional sugarcane and CTC175-A Bt event results in downstream products with no detectable concentrations of heterologous DNA or new protein. These results help in the classification of sugar and ethanol derived from CTC175-A event as pure, chemically defined substances in Brazil and may relieve regulatory burdens in countries that import Brazilian sugar.

## Introduction

Brazil is the largest sugarcane producer and sugar exporter in the world. With an estimated planted area of 9.1 million ha and a total annual yield of 694.54 million tons of sugarcane, Brazil produces an estimated 39.8 million tons of sugar almost entirely devoted to use as a food ingredient. Ethanol fuel production for the domestic and international markets is also an important use of Brazilian sugarcane, representing half of total annual sugarcane yield. Agricultural biotechnology has been used widely in Brazil for almost 20 years in crops such as soybeans, maize, and cotton and recently the Brazilian biosafety authority CTNBio has approved the first biotechnology-derived sugarcane variety for cultivation.

Sugarcane yield is negatively impacted by pests and diseases typically seen in tropical cultivation conditions. A major insect pest impacting Brazilian sugarcane production is the sugarcane borer (*Diatraea saccharalis*). Infestation by this pest has been shown to reduce shoots, tillers, and plant weight, increase lodging, produce drying of young spindle leaves, and allow infections by opportunistic microorganisms, including bacteria and fungi. Yield losses in excess of 10% and a negative impact on sugar quality (increased levels of secondary metabolites such as dextrans and poor color characteristics) are common as a result of borer infestation (Precetti and Téran, 1983; Precetti et al., 1988; Botelho and Macedo, 2002). Centro de Tecnologia Canavieira (CTC), one of the major suppliers of adapted sugarcane germplasms in Brazil, has developed event CTB141175/01-A (abbreviated here as CTC175-A), which expresses the Cry1Ab protein in leaf tissue to control the sugarcane borer. The event also expresses the neomycin-phosphotransferase type II (NptII) protein used as selectable marker during transformation process. The food/feed and environmental safety of event CTC175-A was extensively evaluated by CTNBio, the Brazilian regulatory authority. Vegetative “seed cane” propagation has begun in controlled field conditions leading to commercial sugar production in Brazil in the 2020 timeframe.

Sugar is extracted from sugarcane stalks which are pressed to produce the sugarcane juice. OECD states that the extracted juice has high water content (about 85%) and contains mainly sucrose and reducing sugars (RSs) like glucose and fructose and that its protein content is negligible, around 0.2% of the dry matter (OECD, 2011). Additionally, sugarcane processing involves harsh conditions known to precipitate and denature protein and DNA, leading to the removal of detectable intact plant DNA and protein in raw and refined sugar (Cullis et al., 2014).

**Figure 1:**
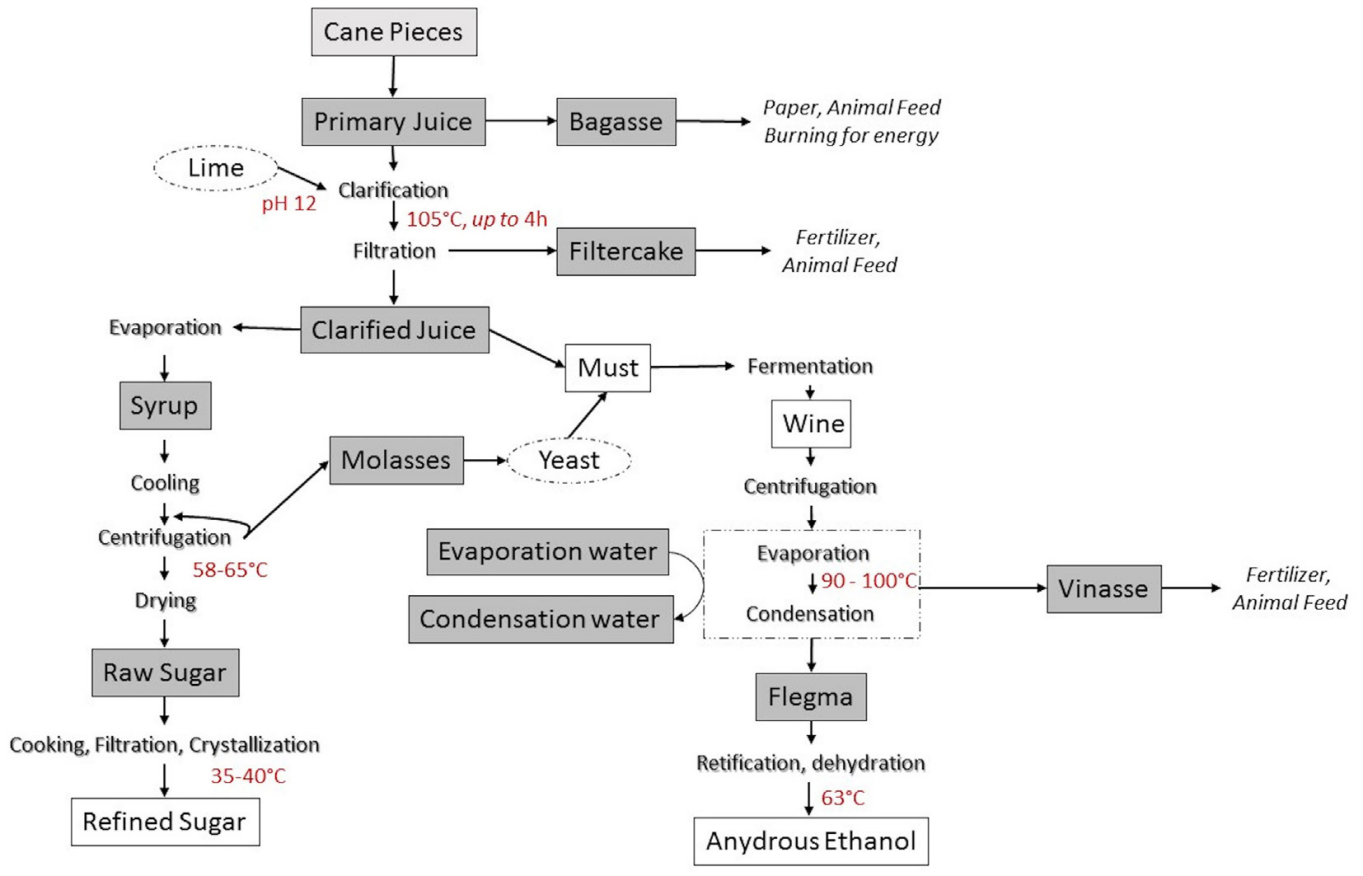
Sugarcane industrial processing and derived food ingredients. Gray boxes indicate sampling points in the process. Lighter gray box (cane pieces) indicates the sampling of leaves.

Industrial production of sugar from sugarcane involves extraction of sugarcane juice, clarification, concentration, crystallization, centrifugation, and sugar drying. The sugar processing can be classified as white sugar production and raw sugar production. White sugar can be produced directly from sugarcane if harsh a clarification step is employed. Alternatively, and more usually, white sugar is produced from an additional refining step of raw sugar (Brokensha, 1998). In raw sugar production, juice is physically extracted from the sugarcane by pressing stalks using either a tandem roller mill or diffuser mill. Cut cane pieces are first shredded, immersed in water and then crushed between sets of rollers to release the primary juice (tandem mill); alternatively, shredded cane is extensively rinsed and percolated with recycling ~80°C water to obtain the primary juice (diffuser mill). The residual fibrous material (Bagasse) is typically dried and used as boiler fuel and the surplus is burned to produce electric energy sold to the public grid. In the second phase, primary juice is filtered and clarified by heating at 105°C for 3 h in the presence of lime (calcium hydroxide) and/or a flocculent to precipitate plant macromolecules (protein, DNA, fiber, etc.). The resulting heavy precipitate, called “mud,” forms which is separated from the juice in the clarifier, and then filtered to produce filter cake which is removed. The resulting clarified juice (14–20°Brix) is concentrated by vacuum evaporation, at an initial temperature of approximately 110°C which then is decreased to 85–90°C, with concomitant increase of vacuum. This evaporation step finishes when syrup of around 65°Brix is produced (Hugot, 1969; Bruijn, 1998). This syrup is concentrated, at 70°C, in a vacuum evaporative crystallizer to produce raw sugar. The first round of sugar crystallization is performed in around 2–3 h but the process can be repeated several times until no more sucrose crystallizes (Hugot, 1969; Bruijn, 1998). The residual liquid called molasses is mixed with sugarcane juice and yeast and fermented to produce ethanol. After recovery of the ethanol the residual fermentation solids are removed by centrifugation to yield vinasse which is typically used as fertilizer. The next process is the refining of the raw sugar to refined sugar which is the final food ingredient (Figure 1).

Therefore, the processes of extraction, raw sugar production, and refining involves multiple steps involving conditions known to denature, precipitate, and eliminate DNA and protein macromolecules found in low concentrations in sugarcane stalks (Cheavegatti-Gianotto et al., 2011; Cullis et al., 2014). As a result, OECD states that sugar is a very purified substance as raw sugar is typically 97–98% sucrose, whereas refined sugar purity is about 99.93% sucrose. The remaining impurities in refined sugar are water, inverted or reducing sugars (glucose and fructose), ash, colored components, and other organic non-sugar compounds (OECD, 2011).

Unlikely other sugarcane producer countries, in Brazil, molasses is almost entirely used for biofuel production, and Brazilian mills do not produce alcoholic beverages, known as “rum,” from this residue. A Brazilian sugarcane spirit, known as *cachaça* or *aguardente*, is produced directly from fresh fermented sugarcane juice, in industrial or artisanal facilities which are distinct from sugarcane mills devoted to sugar and ethanol production. In those facilities, after being extracted, the juice is fermented by yeasts to produce the “wine.” This wine is then boiled in copper stills giving rise to vapors that are then condensed by cooling producing a liquid with high alcohol content (38–54°GL). The liquid obtained in the initial distillation phase is discarded due to the presence of compounds that are more volatile than ethanol. The last fraction of distillation is also discarded due the presence of low volatile substances. In practice, only the middle fraction of distillation, representing 75–85%, is used for consumption. After distillation, this fraction is filtered and consumed directly or after aged in wood barrels. The vast majority of this cachaça production is devoted to domestic market. The steps of boiling and distilling required for cachaça production are likely to remove traces of protein and DNA from the final product.

Due to extremely harsh conditions of sugarcane processing and the resulting purity of those substances, sugar and ethanol produced from all sugarcane, including genetically modified sugarcane, should potentially be classified as “pure substances, chemically defined,” by Brazilian Biosafety Law No. 11.105. One of the requirements for this classification is that the substance should not have the GMO itself, neither heterologous protein/DNA in its final composition. If this classification is to be adopted, these substances are not considered as “GMO derivatives” according to Brazilian Biosafety Law. Additionally, this information is important for importer countries to evaluate the food safety of sugar derived from CTC175-A event. The rationale behind the food risk assessment it that the absence, or presence at extremely low levels of heterologous protein in the article of commerce (sugar) would lead to extremely high consumption safety margins due to none/very low exposure to the heterologous protein. By the scientific point of view, this information, in conjunction with the well established safety of Cry1Ab and NptII proteins, should lessen the safety concerns of using sugar derived from CTC175-A as a food ingredient (Kennedy et al., *in this issue*).

Several experiments, described here, were conducted on conventional sugarcane or on event CTC175-A in Brazil. Specifically, studies evaluated the original expression levels of Cry1Ab and NptII in CTC175-A tissues, and the fate of total protein, Cry1Ab and NptII protein and DNA during processing of event CTC175-A sugarcane. Other studies on conventional sugarcane examined the effects of processing on ribulose-1,5-bisphosphate carboxylase/oxygenase (RuBisCO), DNA, and protein. Results indicate that CTC175-A expresses heterologous proteins in very low levels at the sugarcane juice, the raw material for sugar and ethanol production, and that sugarcane processing degrades/removes protein and DNA leading to the production of sugar and ethanol in which these substances are not identified by conventional detection techniques.

## Materials and Methods

### Sugarcane Event CTC175-A Expression Cassettes and Newly Expressed Proteins

**Figure 2:**
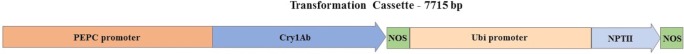
Transformation cassette used to obtain CTC175-A event *via* biolistic transformation of CTC20 sugarcane variety.

Event CTC175-A sugarcane was obtained using biolistic plant transformation, by inserting a DNA fragment containing the expression cassettes for the *cry1Ab* and *nptII* genes into sugarcane variety CTC20, a commercially grown conventional variety cultivated in the Center-South region of Brazil. The DNA fragment used in transformation contains the expression cassettes of the *cry1Ab* gene, which encodes a 648-amino acid *Bacillus thuringiensis* protein, and the *nptII* gene, which encodes 263 amino acid type II neomycin phosphotransferase (Figure 2).

Cry1Ab is a well-studied insecticidal protein, which confers resistance to certain lepidopteran pests including the sugarcane borer (*D. saccharalis*), while NptII is used as a selectable marker used in the transformation process that confers resistance to aminoglycoside-type antibiotics such as neomycin. The expression of the *cry1Ab* and *nptII* genes is regulated by the promoters of the corn Pepcarboxylase gene (PEPC) and the ubiquitin gene of the corn (ubi-1), respectively. Both genes utilize the nopaline synthase terminator (NOS), from *Agrobacterium tumefaciens*. The *cry1Ab* gene present in event CTC175-A corresponds to a synthetic and truncated DNA sequence (Koziel et al., 1992). This sequence had its nucleotides synthetically optimized using preferred codons to enhance expression in corn. The *nptII* gene is derived from the Tn5 transposon of *Escherichia coli* (Fraley et al., 1983).

### Sugarcane Field Agronomic Management

In order to comply with Normative Resolution No. 05 from CTNBio (*Comissão Técnica Nacional de Biossegurança*—Brazilian Technical Biosafety Commission) which requires evaluation of environmental, food, and feed biosafety, and to analyze its phenotypic performance, the event CTC175-A was planted in six locations representative of the crop area of the progenitor cultivar CTC20 in Brazilian Center-South (Paranavaí—Paraná State, Uberlândia—Minas Gerais State, Montividiu—Goiás State; Conchal, Piracicaba, and Jaboticabal—São Paulo State), in the season 2014/2015.

In each location, standard agronomic practices for sugarcane cultivation (soil preparation, fertilization, pest management) were applied evenly throughout the experiment. Treatments (event CTC175-A and the conventional CTC20) were allocated within each block, forming the plots or experimental units. Each plot was represented by four rows of 10 m spaced by 1.5 m adding up an area of 6.0 m × 10.0 m. The experiments were arranged in a randomized complete block-design with 4 replications. In order to assess Cry1Ab and NptII levels at a time representative of harvest and processing to sugar, tissue samples were collected 365 days after planting in field experiments. All experimental fields were conducted under the official CTNBio approvals obtained through compliance with Normative Resolution No. 06.

### Evaluation of Cry1Ab and NptII Expressions in CTC175-A Tissues

In order to evaluate expression levels of proteins Cry1Ab and NptII in CTC175-A event, samples of leaves, stalks, and roots were collected in all replicates from all site experiments and immediately frozen on dry ice until laboratory evaluation. Samples were processed by grinding on dry ice to a fine powder. Protein extractions were performed on representative aliquots of the processed samples. ELISA methodology was used to quantify the proteins in sample extracts.

Cry1Ab protein was extracted from the sugarcane plant tissue samples using the tissue extraction protocol and quantitative assay protocol that follows. An aliquot of each tissue sample was weighed (approximately 15–20 mg) into a 2.0 mL tube. Stainless steel beads were added to each tube. Using buffer ratios of 10:1, an appropriate volume of ELISA extraction buffer (1.5 mL of phosphate-buffered saline with Tween20) was added to each sample. Tissues were pulverized in a Geno Grinder 2010 for approximately 2.5 min at a frequency of 290 *g*. Samples were incubated at 4–8°C for approximately 15 min. Extracts were spun down at ≥12,350 *g* for 10 min at 4°C in a microcentrifuge. Approximately 1.0 mL of the supernatant was collected and placed in a fresh 2.0 mL centrifuge tube. Supernatant from the sample extraction was diluted in deionized water to fall within the range of the standard curve. Remaining supernatants were then frozen at −20°C. The presence of the Cry1Ab protein was detected using a validated ELISA (EnviroLogix Qualiplate Cry1Ab ELISA kit).

Neomycin-phosphotransferase type II protein was extracted from the sugarcane plant tissue samples using the tissue extraction protocol and quantitative assay protocol that follows. An aliquot of each tissue sample was weighed (approximately 45–55 mg for leaf tissue and 190–210 mg for root and internode tissue) into a 2.0 mL tube. Four stainless steel beads were added to each tube. An appropriate volume of ELISA extraction buffer was added to each sample. 1.5 mL of 1× PEB (supplied with kit) was used. Tissues were pulverized in a Geno Grinder 2010 for approximately 2.5 min at a frequency of 290 *g*. Samples were incubated at 4–8°C for approximately 15 min. Extracts were spun down at 12,350 *g* for 10 min at 4°C in a microcentrifuge. Approximately 1.0 mL of the supernatant was collected and placed in a fresh 2.0 mL centrifuge tube. Supernatant from the sample extraction was diluted in 1× PEB to fall within the range of the standard curve. Remaining supernatants were then frozen at −20°C. The presence of the NptII protein was detected using a validated ELISA assay (Agdia NptII ELISA Kit).

Control sample extracts were analyzed concurrently to confirm the absence of plant-matrix effects in ELISA. For each ELISA, a standard curve was generated with known amounts of the corresponding reference protein. Cry1Ab protein standard calibrators at 100, 75, 50, 25, 12.5, 6.25, 3.13, and 0 ng/mL were prepared in deionized water. NptII protein standard calibrators at 20, 15, 10, 5, 2.5, 1.25, 0.625, and 0 ng/mL were prepared in PBST. Calibrators were prepared fresh each day from a working stock solution. The mean absorbance for each sample extract was plotted against the appropriate standard curve to obtain the amount of protein as nanograms per milliliter (ng/mL) of extract. The concentrations were converted to represent the amount of protein as micrograms per gram (μg/g) of tissue by the following formula:

(ng​/​mL)×(dilution factor)×(volume of buffer [mL])/(amount of tissue[g])×1,000.

The predetermined extraction efficiencies were used to adjust the transgenic protein concentrations to the estimated total concentration in the corresponding tissue sample by the following formula:

amount of protein measured from a single extraction (µg​/​g)/extraction efficiency (%).

All calculations, including mean and SD, were performed with Microsoft Excel^®^ 2007 spreadsheet software. All decimal places associated with the concentrations determined for each replicate sample were used in calculation of the mean, where were then rounded to two decimal places for reporting consistency.

### CTC175-A and CTC20-Derived Sugar and Ethanol Production at Laboratory Scale to Evaluate DNA and Protein Loss

In order to comply with Brazilian Biosafety Normatives for regulated genetically modified plant material, one batch of sugar from CTC175-A and one batch of sugar from CTC20 were produced. Mature stalks of CTC175-A event sugarcane and the parental conventional variety CTC20 were collected from all plots of the experiment planted at Piracicaba/SP at 365 days after planting and processed into raw sugar and ethanol using laboratory scale methods (Novello, 2015; Merheb, 2014; Merheb et al., 2016) that mimic the industrial processes used by Brazilian mills. Harvested stalks were immediately transported to the laboratory for sugarcane juice extraction and subsequent processing to collect process fractions including raw sugar.

Approximately 56.0 L of sugarcane juice was extracted from 90 stalks of each variety by shredding and pressing in the laboratory. Leaf, fiber, and sugarcane juice samples were collected from each variety for DNA, protein, and sugar quality analyses. The stalk quality of sugarcane varieties was evaluated by analyses of fiber, starch, brix, dextran, RSs, total RSs, pH, polarization, and purity (Table 1). These characteristics are factors that have a direct impact on the quality of the final products and the yields of the processes (Santos et al., 2012).

**Table 1:** Methodologies used for analyzing characteristics used for sugar classification in Brazilian market.

Characteristic	Methodology	Reference
Starch	Starch—determination in raw sugar	ICUMSA Method GS 1-16 (2009)

Ash	The determination of conductivity ash in raw sugar, brown sugar, juice, syrup, and molasses	ICUMSA – Método GS 1/3/4/7/8-13 (1994)

Color	Determination of solution color of raw sugars brown sugars and colored syrups at pH 7.0	ICUMSA – Method GS 9/1/2/3-8 (2011c)

Dextran	The determination of dextran in raw sugar by a modified alcohol haze method	ICUMSA Method GS 1/2/9-15 (2011b)

Filterability	Método BR-SM-PR-103	Supplemental Methodology S1

Acid Floc	Método BR-SM-PR-420	Supplemental Methodology S1

Alcohol Floc	Método BR-SM-PR-271	Supplemental Methodology S1

RS	Method 32—reducing sugars—determination in raw sugar by the Lane and Eynon method	The Laboratory Manual for Australian Sugar Mills (2001)

Polarization	The determination of the polarization of raw sugar by polarimetry	ICUMSA GS 1/2/3/9-1 (2011a)

Turbidity	Methods of analysis—formazin turbidity standards	ASBC (1976)

Sugars	High-performance liquid chromatography (HPLC)	Sluiter et al. (2006)

The sugarcane juices of CTC175-A and CTC20 were heated to 70°C with constant stirring, immediately after reaching 70°C, the juice was neutralized (pH 7) by adding lime. Following neutralization, juice was further heated to 98–100°C for approximately 2 min, and then transferred to a vessel, containing approximately 3 ppm of anionic polymer flocculant (Flonex 9076)/liter of juice. Following flocculation and decantation, clarified juice (supernatant) was separated from the sludge and samples were collected.

Clarified juice was concentrated from 20 to 65° Brix to generate syrup, using a rotary evaporator. After concentration, this syrup was used in crystallization which was performed using a laboratory reactor (Marconi MA 502), with an 8.0 L internal volume, that was equipped with a helical-type agitator. After the preparation of syrup, 1.0 L of syrup was added in crystallizer to be concentrated from 65 to 84° Brix in vacuum (22in Hg). At this point, 30 g of refined sugar were seeded. Afterward, in the same vacuum, the crystallizer feeding was performed by a controller. When feeding stopped, the crystallizer was in standby for 90 min, and the final evaporation started to be concentrated from 84 to 90° Brix in vacuum. After 6 h, vacuum was removed and the mass was centrifuged and washed with steam. The resultant dense mass of sugar crystals was centrifuged using a laboratory basket centrifuge (Metalúrgica Sueg Ltda), with a capacity of 1.0 kg of crystal sugar per batch (Merheb, 2014; Merheb et al., 2016). In these experiments, approximately 1.0 kg of sugar was produced per crystallization. Following centrifugation, sugar crystals were air-dried for approximately 12 h (Merheb et al., 2016).

Vinasse and Flegma (diluted ethanol) were obtained from the juice in a single cycle batch fermentation performed in triplicate using must composed of sugarcane juice with approximately 160 g/L TRS and 100 g/L fresh PE-2 industrial yeast in a final fermentation volume of 500 mL. Fermentation was performed in an Erlenmeyer flask placed in a shaker (Innova 44, New Brunswick Scientific) at 0.805 *g* and 32°C for 8h. Simple distillation was performed to separate flegma resulting from fermentation from the vinasse, using a distiller (Tecnal Redutec TE–086 alcohol microdistiller). The wine was heated to 90–100°C for 5 min for flegma evaporation and condensation. Vinasse was the distillation residue. For ethanol production, it is necessary to use a distillation column to purify the flegma into ethanol.

All sugar production and sugar analysis were performed in laboratories certified with CQB (*Certificado de Qualidade em* Biossegurança*—*Biosafety Quality Certification) granted by CTNBio according to Normative Resolution No. 01. All personnel working in these activities were trained according the requirements of Normative Resolution No. 02 for contained activities with genetically modified plant material.

#### Compositional Analysis of Sugar Produced in Laboratory

Raw sugars and other common parameters were analyzed at CTC’s laboratories certified with CQB to comply with Brazilian biosafety requirements. The quality of the raw sugar produced from either event CTC175-A or CTC20 conventional was assessed for sugar quality parameters: starch, ash, color, dextran, filterability, acid floc, alcohol floc, RSs, polarization, turbidity, and sugars (sucrose, glucose, fructose). The analytical methodologies used to classify sugar according to Brazilian market are presented in Table 1 and are relevant for sugar classification and placement to specific markets in Brazil (Oliveira et al., 2007a,b).

#### DNA Detection in Laboratory Sugar Fractions

To evaluate the fate of DNA and proteins at the different laboratory processing stages, samples were collected during the processing of sugarcane to raw sugar and ethanol. Both solid samples (leaf, bagasse, and sugar) and liquid samples (primary juice, clarified juice, sludge, syrup, molasses, flegma, and vinasse), were collected from both cultivars (CTC175-A event and CTC20 isoline). The DNA extraction protocol used was based on Aljanabi et al. (1999) with modifications. Solid samples were ground in liquid N_2_ and 5.0 mL of samples were added to 4.0 mL of homogenization buffer (200 mM Tris–HCl, 50 mM EDTA, 2.2 M NaCl, 2% CTAB, 0.06% Na_2_SO_3_, pH 8.0). A detergent solution (2.0 mL of 5% N-lauryl-sarcosine, 2.0 mL of 10% PVP, 2.0 mL of 20% CTAB) was added to the homogenized samples and mixed by inversion for 2–3 min, then incubated for 60 min at 65°C with periodic inversions. After incubation, 10.0 mL of 25:24:1 phenol: chloroform: isoamyl alcohol were added to the samples and mixed by inversion for 2 min. After centrifugation (1,520 *g*, 10 min, 4°C), the supernatant was transferred to a new tube, 10.0 mL of 24:1 chloroform: isoamyl alcohol was added, and mixed by inversion for 2 min. After centrifugation (1,520 *g*, 10 min, 4°C), the supernatant was transferred to fresh tubes, 10.0 mL of isopropanol and 2.0 mL of 6 M NaCl were added, and the tubes were mixed by inversion for 2 min. The samples were then incubated (20°C, 1 h), centrifuged (1,520 *g*, 5 min, 4°C), and the formed pellets were washed two times with 10.0 mL of 70% ethanol. The pellets were dried at room temperature and dissolved in 200 µL of sterile ultrapure water. All DNA samples were quantified in a NanoDrop™ 8000 spectrophotometer (ThermoFisher™). In addition to the total DNA quantification results, all samples from both cultivars (CTC20 and CTC175-A), were also evaluated for the presence of heterologous DNA representing *cry1ab* (GenBank Accession No. AY326434.1) and *nptII* (GenBank Accession No. U00004) genes and the endogenous *ubi1* gene (GenBank Accession No. CA179923.1) genetic elements by TaqMan multiplex analysis (Table 2).

**Table 2:** Primers and probes sequences used to identify exogenous (*cry1Ab* and *nptII*) and endogenous (*ubi*) genes present in CTC175-A by qPCR.

Target	PCR product (pb)	Primer (5′ → 3′)	Primer (5′ → 3′)	Probe
*cry1ab*	102	GTGGACAGCCTGGACGAGAT	GAAGCCACTGCGGAACATG	CCCCTCAGAACAAC
*nptII*	103	GCTCACCCTGTTGTTTGGTGTT	AGCCTCTCCACCCAAGCG	CTTCTGCAGGTCGACTC
*ubi1*	63	ACCATTACCCTGGAGGTTGAGA	GTCCTGGATCTTCGCCTTCA	CTCTGACACCATCGAC

The TaqMan^®^ Multiplex Assay protocol for DNA detection was performed as follows: the reactions were performed in multiplex form to amplify simultaneously one of the two combinations: *cry1Ab*/*ubi1* or *nptII*/*ubi1*. PCR was performed in a 96-well optical plate (“MicroAmp^®^ Fast Optical 96-Well Reaction Plate™,” Life Technologies). The performance of the assays in each processing sample type (e.g., primary juice, bagasse, etc.) was assessed by adding known quantities of the specific DNA for *cry1ab, nptII*, and *ubi1* to processing fractions produced from the CTC20 isoline. All samples were normalized for final DNA concentration of 10 ng/µL and 40 ng were used for each PCR. For samples with DNA concentrations below limit of detection (LOD), 4 µL of DNA solution was used. For positive control samples, two known concentrations of each target gene were used (0.5 and 0.05 ng of DNA).

Reactions were assembled using samples added to a mixture of the following reaction components: 1× Taqman^®^ II Mix Universal Buffer UNG (Applied Biosystems™); forward and reverse primers, each at 500 nM concentration; probes at a final concentration of 200 nM for the multiplex assay cry1Ab/ubi; forward and reverse primer, each at 300 nM concentration; probes at a final concentration of 200 nM for the multiplex assay nptII/ubi; and water in sufficient quantity to make up a final volume of 20 µL. Plates were sealed with optical adhesive film for real-time PCR MicroAmp^®^ (Applied Biosystems™) and PCR was performed in a thermocycler 7500 Fast Real-Time PCR System (Applied Biosystems™) using the following amplification parameters: 2 min at 50°C and 10 min at 95°C, followed by 40 cycles of 15 s at 95°C and 60 s at 60°C. The primers and probes sequences used are detailed in Table 2.

Each PCR amplification curve was examined to determine the presence (+) or absence (−) of DNA by comparing with the respective amplification curve of the positive control. The control DNA concentrations (0.5 and 0.05 ng), were chosen because they represent reliable detection limits of the methodology. Samples that had amplification (Cq) values higher than the positive control at the lowest concentration of DNA (0.05 ng), were considered as non-specific amplifications (<LOD).

#### Detection of Total Proteins in Laboratory Production Fractions

For the extraction and quantification of total proteins, an aliquot (500 µL) of samples taken from the laboratory production of sugar (i.e., leaf, bagasse, juice, filter cake, clarified juice, syrup, molasses, sugar, phlegm, and vinasse) was mixed with 750 µL of protein extraction buffer (0.01 M phosphate buffered saline: 0.138 M NaCl; 0.0027 M KCl, 0.05% TWEEN^®^ 20, pH 7.4), homogenized and centrifuged (10 min, 7.690 *g*). After centrifugation, 600 µL of supernatant was transferred to new tubes and the samples were analyzed for total protein concentration using the Bradford method. A seven-point standard curve with concentrations ranging from 125 to 2,000 mg/mL of bovine serum albumin was produced.

A 96-well flat-bottomed plate was assembled using 10 µL of buffer (null control), 10 µL of each standard (in duplicate) and 10 µL of each sample studied. Bradford solution (200 µL) was added (Bio-Rad Protein Assay Dye Reagent Concentrate™) to each well in a 1:4 ratio (dye:water). The plate incubated on the bench for 5 min and was read on a M2-SpectraMax spectrophotometer (Molecular Devices™).

#### Detection of Cry1Ab and NptII Proteins in Laboratory-Processed Samples

All samples were analyzed for Cry1Ab protein presence using the “QualiPlate™ ELISA Kit for Cry1Ab/Cry1Ac” (ENVIROLOGIX ™) according to the manufacturer’s recommendations. Data were generated using the SpectraMax-M2 Spectrophotometer (Molecular Devices™). A known amount of Cry1Ab protein (~ 0.50 ng) was used to spike a portion of the samples obtained during the production of sugar and ethanol from CTC20-derived samples to obtain an expected final concentration of approximately 3.0 ng/mL of Cry1Ab protein for each sample. A serial dilution curve of the Cry1Ab protein was positioned adjacent to investigated samples in the plate to determine the LOD of the assay. The dilution curve ranged from 50 ng to 3.125 ng/mL of protein.

### Collection of Conventional Sugar Samples From Brazilian Sugarcane

Samples from the industrial processing of conventional sugarcane were collected at two different types of industrial sugarcane mills in Brazil: a tandem roller type (“mill F”) and a diffuser type (“mill C”). Nine sample types were collected from each mill, in the harvest 2016/17: bagasse, primary juice, filter cake, clarified juice, syrup, molasses, vinasse, raw sugar, and flegma. Leaves from CTC20 variety were used as a control sample that does not contain the heterologous DNA and newly expressed proteins. All samples were transported on ice (2–4°C) and stored at −80°C. Leaves from CTC20 variety were used as a control. All samples were stored and transported to CTC on blue ice (2–4°C), and immediately frozen after arriving.

#### Total and RuBisCO DNA Detection in Processing Fractions Obtained From Brazilian Sugarcane Processing Mills

DNA of each fraction was isolated (5.0 mL wet or dry samples in a 50.0 mL conical tube) following the DNA extraction protocol described by Aljanabi et al. (1999). DNA samples were concentrated in an Eppendorf™ Vacufuge™ to a final volume of 0.2 mL before quantification in a Qubit^®^ fluorometer (LifeTechnologies) following the protocol suggested by the manufacturer. DNA samples were also assessed for quality by visualization on ethidium bromide-stained agarose gels.

The sequence of the RuBisCO large subunit of *Saccharum* hybrid cultivar SP80-3280 (GenBank: AE009947.2) was used to design primers for PCR assays (*Saccharum* hybrid cultivar SP-80-3280 chloroplast, complete genome: 119082-120512). Primers were designed to specifically amplify fragments of different sizes. The sequences of the primers and the expected fragment sizes are given in Table 3.

**Table 3:** Primers name, primers sequences, and expected size of amplified fragments.

Combinations	Primer name	Sequence 5′ → 3′	bp
1	SoRcbL_TqM.F	CGCCTCACGGTATCCAAGTT	246
	SoRcbL_R.1	CGGTTTCGGCTTGTGCTT	

2	SoRcbL_TqM.F	CGCCTCACGGTATCCAAGTT	437
	SoRcbL_R.2	TGCTCGGTGAATGTGAAGAAG	

3	SoRcbL_F	CGGAGTACGAAACCAAGGATAC	809
	SoRcbL_R.2	TGCTCGGTGAATGTGAAGAAG	

A dilution curve was prepared using total DNA from sugarcane leaves. Decreasing concentrations ranged from 50 to 0.0125 ng. Reactions were prepared in a final volume of 25 µL using the following reaction components: 1× DreamTaq Green PCR Master Mix (Thermo Scientific), 0.2 μM of each primer, and 5 µL of genomic DNA of known concentrations, to have five different points of dilution curve for each tested pair of primers (50, 25, 12.5, 0.125, and 0.0125 ng).

PCRs were performed on the Proflex^®^ thermal cycler (Applied Biosystem), according to the following step-cycle program: initial denaturation step at 92°C for 2 min; 30 cycles consisting of denaturation at 92°C for 30 s, annealing at 60° for 40 s (primers combinations 1, 2, and 3) or annealing at 50°C (primers combinations 4 and 5), and extension at 72°C for 60 s; final extension step at 72°C for 7 min. After the amplification, PCR products were electrophoresed on 2% agarose gels in 1× TBE solution, stained with 0.4 µg/mL ethidium bromide, visualized under ultraviolet light (UV), and registered with transilluminator and software L.PIX Loccus Biotechnology.

Reactions were performed in 25 µL final volume with variable amounts of template. For fractions from which DNA was quantifiable, serial dilutions were prepared; seven points (10, 5, 0.5, 0.25, 0.025, 0.0025, and 0.00125 ng) were amplified in each PCR. When DNA concentrations from fractions were below the LOD (<LOD), an arbitrary volume of sample was added to the amplification reactions. Thus, for raw sugar and flegma from the tandem roller mill samples and, raw sugar, flegma, and clarified juice samples from diffuser mill 5, 2.5, 1.25, and 0.5 µL of template were used. DNA from leaf was used as positive control (2.5 ng). In parallel, an aliquot of the same matrix of each sample was spiked with DNA from sugarcane leaves. Again, a serial dilution was done to have an input of approximately 2, 1, 0.1, 0.01, and 0.001 ng of DNA in each reaction. For raw sugar and flegma from both types of mills and for clarified juice from diffuser mill, the dilution curve consisted of 12.5, 6, 3, and 1 pg were used. Reaction components and the cycling program followed as described previously, using 28 amplification cycles. PCR products were electrophoresed on 2% agarose gels.

#### Total Protein Detection in Processing Fractions Obtained From Brazilian Sugarcane Processing Mills

After freezing, 6.0 mL of each sample was aliquoted in six tubes (1.0 mL each) and lyophilized for 6 days at −60°C with exception of flegma. Samples from leaves and bagasse were further ground to a homogeneous powder and protein extraction was performed as described by Cullis et al. (2014) with minor modifications. Lyophilized samples from individual tubes combined into a 15-mL polypropylene tube and prepared as solutions, where former solid samples (leaves, bagasse, and filter cake) were dissolved at a 3% (w/v) in water. Similarly, primary juice, clarified juice, syrup, molasses, raw sugar, and vinasse were dissolved in a 10% (w/v) in water. Flegma was prepared as a solution 20% (v/v) in water. This procedure was done in duplicate, with half of the mill’s fractions (controls) spiked with 1,000 ng of total protein, previously extracted from sugarcane leaves and quantified using microplate “Micro BCA protein assay” (ThermoFisher). In the case of raw sugar and flegma, aliquots were spiked with 10 µg of total protein. All solutions were adjusted to 1% sodium dodecyl sulfate (SDS) + 10 mM dithiothreitol + 10 mM Tris–HCl pH 7.5 + 0.5 mM PMSF (SDS extraction buffer) and placed at 65°C for 60 min with occasional mixing by inversion. Tubes were centrifuged (6,500 *g*) for 15 min at room temperature. To 2 mL of the supernatant, 3 mL of 1% sodium deoxycholate were added followed by 1.25 mL of 50% trichloroacetic acid. After mixing and incubating on ice for 15 min, the tubes were centrifuged at 6,500 *g* for 15 min at 4°C, supernatant was discarded, and the pellet drained for 5 min. Pellets were then washed with 1.5 mL of acetone by vigorous mixing for 15 s followed by incubation at 25°C for 15 min with occasional mixing. Samples were placed on ice for 10 min, centrifuged, the supernatants removed, and the tubes drained at room temperature. Next, 1.5 mL of 85% acetone was added with mixing, and the tubes were centrifuged, drained, and dried at 37°C for 15 min. The precipitate was dissolved in 0.5 mL of 0.5% SDS + 10 mM Tris–HCl pH 7.5 at 65°C for 20 min, with occasional mixing. Assuming 100% of recovery, the concentration of total proteins in spiked samples per microliter of resuspended extract, should be 2 ng/µL. Protein content was determined in both, original mill fractions and spiked mill fractions (controls), using the microplate Micro BCA protein assay (ThermoFisher) as recommended by the manufacturer. SDS-PAGE was used to check extracted protein quality of each sample. About 2–20 µg of total protein were diluted in sample buffer (2× Laemmli Buffer, Biorad, USA) and denatured at 100°C for 5 min. Proteins were separated under denaturing conditions on a 4–20% polyacrylamide gel (Mini-PROTEAN TGX, Biorad, USA) ready to use. At the end, protein gels were stained with Coomassie Brilliant Blue (EZBlue, Sigma, USA).

#### RuBisCO Protein Detection

ELISA was performed according the manufacturer’s recommendations (“Plant RuBisCO ELISA Kit”—Catalog # MBS705973—MyBioSource). The detection range described in the kit’s protocol is 3.12–800.0 µg/mL. Thus, using the standard sample solution supplied with the commercial kit a standard curve was prepared with five points of known RuBisCO concentration (μg/mL). The protein sample eluent (0.5% SDS + 10 mM Tris–HCl) serves as the zero standard and the curve blank. The total protein samples and the buffer used to elute the protein after extraction were diluted to 1/2, 1/5, 1/10, and 1/100. The mean absorbance of each buffer dilutions was used as a blank for each sample dilution. The concentration read from the standard curve was multiplied by the dilution factor. The optical density of each well was determined using a microplate reader (SpectraMax, Molecular Devices, USA) set to 450 nm with wavelength correction of 540 and 570 nm.

## Results and Discussion

### Expression of Cry1Ab and NptII on Tissues of CTC175-A Events

The construct used to obtain CTC175-A event was designed to express Cry1Ab preferentially in leaves, where the sugarcane borer lays its eggs and starts its development. The promoter used to drive Cry1Ab expression, PEPC, is known to confer preferential expression in photosynthesizing tissues (Harrison et al., 2011). Therefore, as expected, the highest concentrations of Cry1Ab were found in leaf tissue in all evaluated sites; much lower levels were found in roots and stalks [below the limit of quantification (LOQ) of ≤235 ng/g FW tissue] (Table 4).

**Table 4:** Concentration of Cry1Ab protein in tissues of CTC175-A event evaluated *via* ELISA (*n* = 4).

Site	Tissue	Cry1Ab (μg/g FW)	SD	SE
Jaboticabal	Stalk	<LOQ	–	–
	Leaf	64.01	9.59	4.8
	Root	0.40/0.55/0.58^a^	–	–

Montividiu	Stalk	<LOQ	–	–
	Leaf	40.7	10.5	5.2
	Root	0.81/0.83/0.87^a^	–	–

Piracicaba	Stalk	<LOQ	–	–
	Leaf	64.9	11	5.5
	Root	<LOQ	–	–

Conchal	Stalk	0.31^b^	–	–
	Leaf	63.4	6.7	3.4
	Root	0.67/0.70^c^	–	–

Uberlândia	Stalk	<LOQ	–	–
	Leaf	67.9	20.6	10.3
	Root	<LOQ	–	–

Paranavaí	Stalk	0.37^b^	–	–
	Leaf	29.6	3.1	1.6
	Root	0.84^b^	–	–

*Limit of quantification for leaf, stalk, and root tissues: LOQ ≤ 0.235 µg/g*.

*^a^Three repeats above LOQ*.

*^b^One repeat above LOQ*.

*^c^Two repeats above LOQ*.

In Table 4, Cry1Ab expression values in leaves were expressed as mean of four repeats, with their respective SE and SDs, for each site. As for root and stalk tissues, expression of Cry1Ab is much lower and some repeats data were below of LOQ of ELISA assay. When at least one repeat had measurement above LOQ, data of Cry1Ab expression for those repeats above LOQ were reported directly without any statistical analysis. It was only possible to detect Cry1Ab expression in stalks, the raw material for sugar and ethanol production, for only one repeat of each site, Conchal (310 ng/g FW tissue) and Paranavaí (370 ng/g FW tissue).

The expression of NptII on event CTC175-A was solely required for selecting transformed events during the transformation process. The *Ubi* promoter driving expression of the *nptII* gene in the transformation cassette is known to be expressed in rapidly dividing tissues (Christensen et al., 1992). Therefore, results show low levels of NptII expression in leaves 0.07–0.16 µg/g FW and even lower expression in roots ranging from below the LOQ (<34 ng/g FW) to 70 ng/g FW. NptII expression was at or below detectable levels in sugarcane stalks (<34 ng/g FW) (Table 5).

**Table 5:** Concentration of NptII protein in tissues of CTC175-A event evaluated *via* ELISA (*n* = 4).

Site	Tissue	NptII (μg/g FW)	SD	SE
Jaboticabal	Stalk	<LOQ	–	–
	Leaf	0.15	0.02	0.01
	Root	<LOQ	–	–

Montividiu	Stalk	0.02^a^	–	–
	Leaf	0.08	0.02	0.01
	Root	0.04/0.04^b^	–	–

Piracicaba	Stalk	0.01^a^	–	–
	Leaf	0.16	0.02	0.01
	Root	0.07	0.01	0.01

Conchal	Stalk	0.02^a^	–	–
	Leaf	0.13	0.03	0.02
	Root	0.05	0.01	0.01

Uberlândia	Stalk	0.01^a^	–	–
	Leaf	0.03	0.07	0.03
	Root	0.04^a^	–	–

Paranavaí	Stalk	0.02/0.01^b^	–	–
	Leaf	0.13	0.05	0.02
	Root	0.06/0.24^b^	–	–

*Limit of quantification for leaf tissues: LOQ ≤ 0.034 µg/g. Limit of quantification for stalk and root tissues: LOQ ≤ 0.0094 µg/g*.

*^a^One repeat above LOQ*.

*^b^Two repeats above LOQ*.

At Table 5, as for Cry1Ab, NptII expression values in leaves were expressed as mean of four repeats, with their respective SEs and SDs, for each site. As for root and stalk tissues, expression of NptII is much lower and some repeats data were below LOQ. When at least one repeat had measurement above LOQ, it was decided to report data of NptII expression for those repeats above LOQ, without any statistical analysis.

These results indicate that sugarcane stalks, which are the raw material for sugar and ethanol production, presents originally low levels of heterologous protein expression. This is not surprising due to the nature of promoters used to drive gene expressions and the fact that sugarcane naturally presents negligible protein levels in stalks (OECD, 2011). In fact, the search for promoters that ensures high protein expression levels in sugarcane stalks is still a scientific challenge (Damaj et al., 2010).

### Composition of Sugar Obtained From the Laboratory Processing

The quality of the harvested sugarcane for industrial processing is an important consideration for processing mills because stalk quality directly affects the sugar and ethanol production potential (Garcia, 2012; Santos et al., 2012; Santos and Borem, 2013). One batch of sugarcane juice for CTC175-A event and one for CTC20 cultivar were prepared, therefore, the values presented at Tables 6 and 7 should be evaluated as single measures and not as estimates of quality parameters of juices and sugars produced from CTC175-A and CTC20. Despite this, the results of quality parameters of both juices were within the recommended range for these sugarcane analytes (Table 6), confirming that the raw material used for the laboratory production for sugar and ethanol in this study was acceptable. This procedure is commonly used by Brazilian mills to evaluate and to pay according to the content of sucrose (Pol% juice) in sugarcane (Bruijn, 1998). The other parameters were evaluated for key sugarcane processing steps. Overall, juices produced in laboratory scale resembled juice ordinally processed in Brazilian mills.

**Table 6:** Results of sugarcane quality analysis.

Characteristic	Unit	Recommended values (Santos and Borem, 2013)	CTC20	CTC175-A
Fiber	% sugarcane	10–13	10.72	10.41
Starch	mg/kg	<1,000	875	948
Brix	% juice	>14	21.02	20.84
Dextran	mg/kg	<10	<10	<10
RS	% juice	<0.8	0.47	0.47
TRS	% juice	>15	20.54	20
pH	–	4.8–5.8	5.4	5.3
Pol	% juice	>14	19.43	19.26
Purity	% juice	>85	92.44	92.42

*Single batch results (*n* = 1)*.

In Brazil, it is the usual practice to employ COPERSUCAR specifications to classify sugars for different industrial applications. According to the physicochemical parameters evaluated (Table 7), sugar produced from both the event CTC175-A and CTC20 conventional were classified as “Type 3C” according to the classifying parameters: conductometric ashes ≤ 0.1%, color ICUMSA ≤ 400 and sugar content (Pol Z) above 99.5%, published by COPERSUCAR (2015). The high quality of Type 3C sugar produced, which technically can be labeled as “white sugar,” is not surprising even though the sugar production method employed here was the typical method for production “raw sugar.” This higher grade result can also be obtained in real world sugar production when the quality of starting sugarcane juice is high (Santos et al., 2012).

**Table 7:** Physicochemical parameters of example raw sugar lots produced from control cultivar CTC20 and CTC175-A including relevant copersucar raw sugar classification specifications.

Characteristic	Unit	CTC20	CTC175-A	Type 3C
Starch	mg/kg	294	207	–
Conductometric ash	% m/m	0.02	0.03	max 0.1
ICUMSA color (MOPS)	IU	200	246	max 400
Dextran	mg/kg	<10	<10	–
Filterability	mL–min	45–5	43–5	–
Acid beverage floc	–	Negative	Negative	–
Alcohol floc	Abs	0.055	0.083	–
RS	% m/m	<0.06	<0.06	–
Polarization	Z	99.74	99.67	min 99.5
Turbidity	NTU	15	40	–

*Single batch results (*n* = 1)*.

### Detection of *cry1Ab* and *nptII* DNA Sequences of Fractions of Laboratory Processing of CTC175-A

The results of detection of gene sequences of DNA (*cry1Ab, nptII*, and endogenous *ubi*) in samples from the laboratory fractions of sugarcane processing showed the presence of endogenous DNA (*ubi* gene) in leaf, bagasse, primary juice, and in the precipitated fraction of clarification process filter cake, both from the laboratory processing of event CTC175-A and CTC20 (Tables 8 and 9). The molasses fractions from CTC20 but not from CTC175-A event also showed the presence of the *ubi* gene (Tables 8 and 9). The vinasse sample showed detection of *ubi* gene at levels equivalent to presence of a DNA concentration below 0.05 ng (positive control) (Table 8) and for the juice processed from CTC175-A (Table 9). Flegma also showed detection of *ubi* gene at levels equivalent to presence of a DNA concentration below 0.05 ng (positive control) (Table 9).

**Table 8:** Results of gene amplification of *ubi* (endogenous gene) and *cry1Ab* in samples collected during the laboratory sugarcane processing to produce sugar and ethanol from CTC20 and CTC175-A.

	CTC20 + 0.05 ng DNA (*cry1ab*)		CTC20		CTC175-A	
	*ubi*	*cry1ab*	*ubi*	*cry1ab*	*ubi*	*cry1ab*
Leaf	+	+	+	−	+	+
Bagasse	+	+	+	−	+	+
Primary juice	+	+	+	−	+	+
Filter cake	+	+	+	−	+	+
Clarified juice	+	+	−	−	−	−
Syrup	+	+	−	−	−	−
Molasses	+	+	+	−	−	−
Sugar	+	+	−	−	−	−
Flegma	+	+	−	−	−	−
Vinasse	+	+	<LOD	−	<LOD	<LOD
Evaporation water	+	+	−	−	−	−
Condensation water	+	+	−	−	−	−

*Samples obtained from cultivar CTC20 were intentionally spiked with 0.5 and 0.05 ng of DNA from CTC175-A event. +: presence; −: absence; <LOD: the amplification curve shows cq later than the same sample at a concentration of 0.05 ng of total DNA per reaction*.

**Table 9:** Results of gene amplification of *ubi* (endogenous gene) and *nptII* in samples collected during the sugarcane laboratory processing to produce sugar and ethanol from CTC20 and CTC175-A.

	CTC20 + 0.05 ng DNA (*nptII*)		CTC20		CTC175-A	
	*ubi*	*nptII*	*ubi*	*nptII*	*ubi*	*nptII*
Leaf	+	+	+	−	+	+
Bagasse	+	+	+	−	+	+
Primary juice	+	+	+	−	+	+
Filter cake	+	+	+	−	+	+
Clarified juice	+	+	−	−	<LOD	<LOD
Syrup	+	+	−	−	−	−
Molasses	+	+	+	−	−	−
Sugar	+	+	−	−	−	−
Flegma	+	+	< LOD	−	<LOD	−
Vinasse	+	+	−	−	+	−
Evaporation water	+	+	−	−	−	−
Condensation water	+	+	−	−	−	−

*Samples obtained from cultivar CTC20 were intentionally spiked with 0.5 and 0.05 ng of DNA from CTC175-A event. +: presence; −: absence; <LOD: the amplification curve shows cq later than the same sample at a concentration of 0.05 ng of total DNA per reaction*.

The results for amplification of *cry1Ab* gene from CTC175-A event were completely concordant with the results of *ubi* amplification in CTC175-A and CTC20, revealing positive amplifications for the less processed fractions (leaf, bagasse, and primary juice) and the precipitated residue filter cake (Table 8). Additionally, vinasse from CTC175-A also showed detection of *cry1Ab* gene at levels equivalent to presence of a DNA concentration below 0.05 ng, as for *ubi* gene from CTC175-A and CTC20. All samples spiked with 0.05 ng of DNA from *cry1Ab* gene showed expected amplifications, ensuring the absence of matrix negative influence on DNA amplifications, at least at the level of quantification of this assay. These results clearly show that the *cry1Ab* DNA was degraded and/or removed in the juice clarification step, and subsequent downstream fractions, including raw sugar, did not contain detectable levels of *cry1Ab* gene DNA.

The results of detection of *nptII* gene are similar to those for *cry1Ab* detection (Table 9). There was DNA detection in all processing samples spiked with appropriated amount to detect 0.05 ng of *nptII* DNA (positive controls) showing that the gene, if present could be amplified and detected in each fraction. Samples obtained from byproducts of CTC20 processing detected DNA presence only for the *ubi* gene in unprocessed samples (leaves) or less processed (bagasse, juice, and filter cake), except for molasses which also showed amplification. The flegma sample showed detection equivalent to a DNA concentration below 0.05 ng (Table 9). Samples from of event CTC175-A event showed DNA presence for both the genes (*ubi* and *nptII*) in samples without processing (leaf) or minimal processing (bagasse, juice, and filter cake) as described for detecting the endogenous gene in the CTC20 cultivar (Table 9).

These results obtained by TaqMan assay are consistent with the findings of Cullis et al. (2014) who also found a dramatic reduction in total sugarcane DNA quantity upon production of the clarified juice, the common starting material for production of raw sugar and ethanol production. No heterologous DNA was detected by PCR amplification in the final products raw sugar or flegma (the starting material for ethanol production).

### Detection of Total Proteins in Fractions of Laboratory Production of Sugar and Ethanol

Protein quantification methodology was effective in detecting measurable amounts of protein (above LOD) for samples of leaves, bagasse, and primary juice from event CTC175-A and the CTC20 conventional (Table 10) collected in the laboratory-scale preparation of sugar and ethanol. The values presented at Table 10 should be evaluated as single measures and not as estimates of total protein content in fractions of processing of CTC175-A and CTC20 because there was only one preparation for each material. It was not possible to detect measurable total proteins in samples after juice clarification for both, CTC175-A and CTC20, indicating this process leads to either protein degradation or precipitation.

**Table 10:** Total protein quantification after Bradford extraction in fractions of laboratory sugarcane processing.

Sample	Total protein (mg/mL)	
	CTC175-A	CTC20
Leaf	0.64	0.79
Bagasse	0.05	0.02
Primary juice	0.06	0.12
Clarified juice	Not detected	Not detected
Filter cake	Not detected	Not detected
Syrup	Not detected	Not detected
Molasses	Not detected	Not detected
Sugar	Not detected	Not detected
Phlegma	Not detected	Not detected
Vinasse	Not detected	Not detected
Evaporation water	Not detected	Not detected
Condensation water	Not detected	Not detected

*Single batch results (*n* = 1)*.

### Detection of Cry1Ab in Raw Sugar Produced in Laboratory Scale

Cry1Ab protein was detected in leaves, bagasse, and primary juice produced in laboratory from event CTC175-A sugarcane. The remaining samples that have undergone various chemical and/or heat treatments during the manufacturing process of obtaining sugar and alcohol, as well as the final processed products (sugar and flegma) showed no detectable Cry1Ab protein (Table 11). As expected, samples from CTC20 cultivar did not contain detectable Cry1Ab protein whereas samples from CTC20 spiked with Cry1Ab protein showed detection of protein in all cases, demonstrating lack of matrix interference with the detection assay. Molasses and vinasse samples were reported as <LOD because, although there was antibody reaction for these samples, the OD reading was below the lowest point of the dilution curve (3.125 ng).

**Table 11:** Presence (+) and absence (−) of Cry1ab protein in fractions samples from the industrial processing of CTC20 and CTC 175-A Varieties, using ELISA methodology.

Samples	CTC175-A	CTC20	CTC20 + Cry1ab
Leaf	+	−	+
Bagasse	+	−	+
Primary juice	+	−	+
Clarified juice	−	−	+
Filter cake	−	−	+
Syrup	−	−	+
Molasses	−	−	<LOD
Sugar	−	−	+
Phlegma	−	−	+
Vinasse	−	−	<LOD
Evaporation water	−	−	+
Condensation water	−	−	+

*<LOD: below protein detection level per the dilution curve of known concentrations*.

### Detection of DNA, Total Proteins, and RuBisCo in Fractions of Industrial Processing

The SDS-PAGE evaluation from samples from diffuser mill revealed a protein smear in all samples (Figure S1A in Supplementary Material). It was possible to detect smeared proteins in samples of leaves, primary juice and filter cake. It was not possible to detect total proteins from clarified juice and downstream samples (syrup, molasses, and raw sugar) (Figure S1A in Supplementary Material). It was not possible to detect proteins in raw sugar samples (Figure S1B in Supplementary Material) produced in tandem roller and diffuser mills.

The evaluation of total DNA from samples from the Brazilian mill processing fractions revealed that the quantity of extracted total DNA ranged from 173 ng/µL in bagasse to 1.36 ng/µL in clarified juice from samples derived from the tandem roller mill (Table 12). Raw sugar and flegma were below the LOD of Qubit^®^ Quantitation Assay Kit (0.2 ng). Samples from the diffuser mill presented as much as 16.6 ng of DNA per μL of bagasse to approximately 1 ng/µL in the molasses fraction. Samples from raw sugar, flegma, clarified juice, and syrup were below of LOD of Qubit^®^ reagents (Table 12). Samples from sugarcane leaves yielded as much as 880 ng/µL of DNA.

**Table 12:** DNA quantification for each processing fraction sample from two types of mills.

DNA quantification		
Sample	Tandem roller mill (ng/μL)	Diffuser mill (ng/μL)
Leaves CTC20	880.0	
Bagasse	173	16.6
Primary juice	11.5	6.4
Clarified juice	1.36	<LOD
Filter cake	26.8	16.5
Syrup	1.84	<LOD
Molasses	2.69	0.93
Vinasse	37.2	26.8
Raw sugar	<LOD	<LOD
Flegma	<LOD	<LOD

*Single batch results (*n* = 1)*.

The values presented at Table 12 should be evaluated as single point estimates of DNA content in fractions from tandem roller and diffuser Brazilian mills. Overall, samples from both mills showed a trend of decreasing DNA concentration throughout the processing steps. The final products of processing (raw sugar and flegma) did not presented DNA above the LOD of this assay for both types of mills (Table 12).

Primer combinations 1 and 2 (Table 3) were used to obtain results of RuBisCo DNA detection with samples from both types of mills. The results from diffuser mill showed RuBisCo DNA detection for all samples except clarified juice, raw sugar, and flegma. The positive controls (i.e., spiked samples) yielded amplification down to the 0.01 ng level for all samples except vinasse, which could only be observed at the 0.1 ng level. Spiked samples of clarified sugar, raw sugar and flegma supported amplification at 3 pg of DNA. The results from tandem roller mill fractions were similar with those found from diffuser mill samples and indicates that RuBisCo DNA can be detected in all processing fractions except raw sugar and flegma (Table 13). The positive controls (i.e., spiked samples) yielded amplification down to the 0.01 ng level for all samples except vinasse, which could only be observed at the 0.1 ng level. Spiked samples of raw sugar and flegma supported amplification at 3 pg of DNA. Therefore, these results are concordant in not identifying RuBisCo DNA in raw sugar and flegma.

**Table 13:** RuBisCO DNA detection in fractions collected during industrial processing of sugar and ethanol production from two types of sugarcane mills.

	Diffuser mill		Tandem roller mill	
	Fraction samples	Fraction samples spiked (+)	Fraction samples	Fraction samples spiked (+)
Bagasse	+	+	+	+
Primary juice	+	+	+	+
Filter cake	+	+	+	+
Clarified juice	<LOD	^a^	+	+
Raw sugar	<LOD	^a^	<LOD	^a^
Flegma	<LOD	^a^	<LOD	^a^
Vinasse	+	^b^	+	^b^
Leaf	+		+	

*+: positive detection; <LOD: below of limit of detection; spiked (+): samples contaminated with DNA detection of 0.01 ng (nanogram)*.

*^a^Positive amplification for samples contaminated with DNA 3 pg of DNA*.

*^b^Positive amplification for samples contaminated with DNA 100 pg of DNA*.

The values obtained for Total protein and RuBisCo quantification in processing fractions of tandem roller and diffuser Brazilian mills should be evaluated as single point estimates. In samples collected from mills, BCA protein quantification demonstrated that most of protein content present at sugarcane juice is eliminated in the precipitated filter cake (Table 14) resulting in a protein content in clarified juice at least two orders of magnitude lower than in primary juice. The final protein content in raw sugar and in flegma is minimal (Table 14).

**Table 14:** Total protein quantification using BCA in fraction collected from two types of mills.

Sample	Diffuser mill total protein (μg/mL)	Tandem roller mill total protein (μg/mL)
Leaf	~1.500	
Bagasse	133	267
Primary juice	1.319	1.237
Filter cake	623	933
Clarified juice	13	54
Syrup	8	24
Molasse	88	418
Raw sugar	4	10
Flegma	1	9
Vinasse	581	284

*Single batch results (*n* = 1)*.

**Figure 3:**
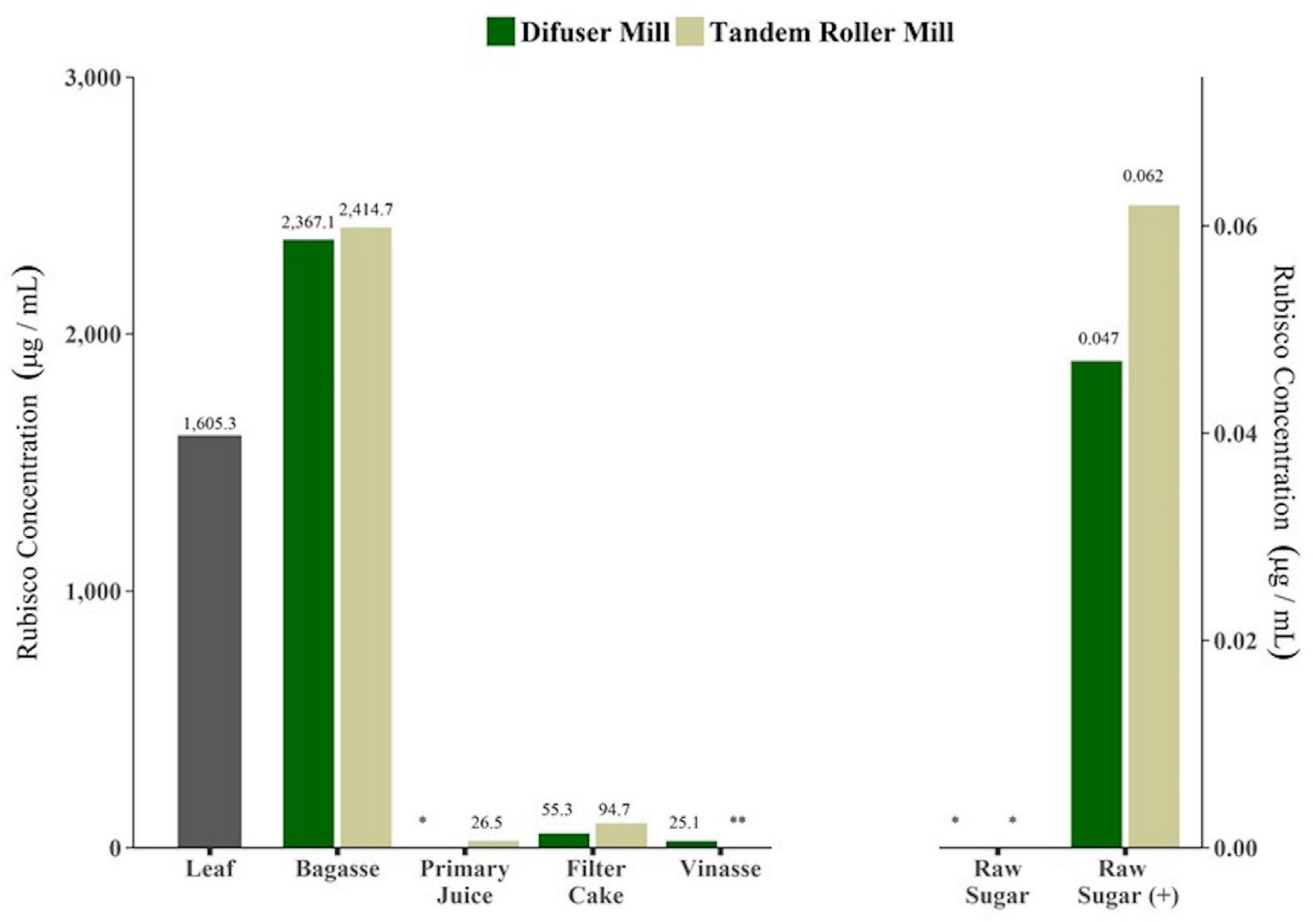
Ribulose-1,5-bisphosphate carboxylase/oxygenase (RuBisCO) protein detection in by product samples collected during industrial process for sugar and alcohol production, from two types of sugarcane mills, by ELISA assay. Single batch results (*n* = 1). *: below limit of detection (LOD). **: not analyzed. Raw sugar (+): samples of raw sugar spiked with 10 µg of total protein before extraction.

Results of RuBisCo quantification were concordant with evaluation of BCA total protein quantification. Figure 3 shows the results for ELISA assay for RuBisCO concentrations in samples from fractions obtained from commercial tandem and diffuser mills processing plants. Leaf and bagasse are far above the range of quantification (3.12–800 µg/mL), therefore are not accurate estimates. RuBisCO protein was also detected in samples of primary juice, filter cake and vinasse. In the case of primary juice, it was not possible to quantify RuBisCO in samples from diffuser mill. Samples of vinasse from tandem roller mill were not analyzed. As expected, the concentrations of RuBisCO were below the LOQ in samples of raw sugar produced from both type of mills. In samples of raw sugar spiked with 10 µg of RuBisCO protein before extraction, ELISA was sensitive enough to detect as little as 0.05 or 0.06 µg/mL of RuBisCO, confirming that the protein could have been detected at those levels if it was present in raw sugar.

## Conclusion

The results presented in this study demonstrates that event CTC175-A presents very low expression of Cry1Ab and nptII proteins in stalks, the raw material for sugar and ethanol production. This result is in agreement with the design of the DNA cassette used to obtain this event, that was constructed to drive high levels of Cry1Ab in leaves. Besides, several assays of fractions of laboratory processing strongly suggests that total DNA, total protein, heterologous DNA and Cry1Ab protein are degraded during processing, leading to concentrations that are not easily detected by commonly used methodology employed to evaluate the presence of GMOs or GMOs derivative in food/feed.

Three lines of evidence clearly establish that raw sugar does not contain detectable levels of either the inserted heterologous DNA or expressed proteins. First, published studies of total DNA and protein loss during stalk processing to refined sugar showed levels of <1 pg total DNA/g refined sugar and ~1 μg total protein/g refined sugar (Cullis et al., 2014). Given these extremely low detection levels for total DNA and protein, it is expected that the small quantities of heterologous DNA and newly expressed protein would also be no detectable. Second, studies presented herein (Tables 11–14; Figure 3), tracked the concentrations of total protein and RuBisCO protein during stalk processing to refined sugar in two types of commercial processing plants in Brazil. RuBisCO is the single most abundant stalk protein (up to 30% of total plant protein) with very high DNA copy number. These studies confirmed the results of Cullis et al. (2014) that the extent of protein loss during processing is at least three to four orders of magnitude (2–5 mg of total protein per gram of cane preextraction and 0.75–1.875 µg total protein in raw sugar derived from 1 g of cane). Finally, and most importantly, studies with new event CTC175-A sugarcane stalks, clarified juice, molasses, and raw sugar showed no detectable levels of Cry1Ab protein (by ELISA, <235 ng/g FW tissue) in stalks or processed fractions. Similarly, no heterologous DNA was detected in clarified juice and downstream products including raw sugar. These results are in agreement with the results of other studies that investigated the degradation of specific DNA fragments inserted into genetically modified sugarcane (NptII) or glyphosate-resistant sugar beet (CP4 EPSPS) that reported the complete elimination of the inserted DNA during processing to refined sugar (Klein et al., 1998; Oguchi et al., 2009; Joyce et al., 2013).

In conclusion, results reported here demonstrate lack of detectable protein and DNA from CTC175-A at reasonable levels of sensitivity in processing fractions of sugarcane, including raw sugar, and are in alignment with previous studies reported in Cullis et al. (2014) and Joyce et al. (2013) on sugarcane. Detectability and quantification of these analytes (proteins in particular) are directly relevant to the globally accepted comprehensive safety assessment strategy on biotechnology-derived crops. Quantification forms the underpinning for the exposure component of the risked-based safety assessment; low/no exposure to the heterologous proteins expressed in CTC175-A in conjunction with the extensively reviewed hazard assessment data on those proteins showing no measurable toxicity to humans, animals, or the environment, support the safety conclusions on CTC175-A. Currently, there are no regulations specific to sugarcane related to DNA or protein detection; this work seeks to establish viable parameters to determine levels of exposure to potential toxicants. It is though publications such as this that government and industry standards can be derived and justified.

## Ethics Statement

All manipulation of genetically modified organisms and their derivatives were strictly performed according to Brazilian Biosafety Law 11.105 and CTNBio regulations and required approvals.

## Author Contributions

ACG, RL, and WO conceived the study; AG, GM, MS, and TF performed experiments; ACG, RL, AG, and MS analyzed the data; ACG, AG, GM, RL, and WO wrote the manuscript; DO, GM, and MS provided supportive information.

## Conflict of Interest Statement

AG, AG, DO, GM, MS, TF, and WO are employees of CTC, which is developing products related to the research being reported. RL was a consultant to CTC on biosafety studies of products related to the research being reported. The reviewer RW and handling editor declared their shared affiliation.

## Supplementary Material

Figure S1 SDS-PAGE gel of protein in fractions of Brazilian mills. M, molecular 838 Marker; BG, bagasse; PJ, primary Juice; FC, Filter Cake; CJ, Clarified juice; SY, syrup; MO, 839 molasses; X, empty lane, L, leaf; RW, Raw sugar. Number following letters indicates spiking of 840 correspondent amount (in μg) of total total protein before protein extraction.

Table S1 Supplemental methodology.
