# Supplementary figures and images for: Lack of Detection of Bt Sugarcane Cry1Ab and NptII DNA and Proteins in Sugarcane Processing Products Including Raw Sugar
Source: Front Bioeng Biotechnol. 2018 Mar 27;6:24. doi: 10.3389/fbioe.2018.00024 (PMC5880997; doi:10.3389/fbioe.2018.00024)

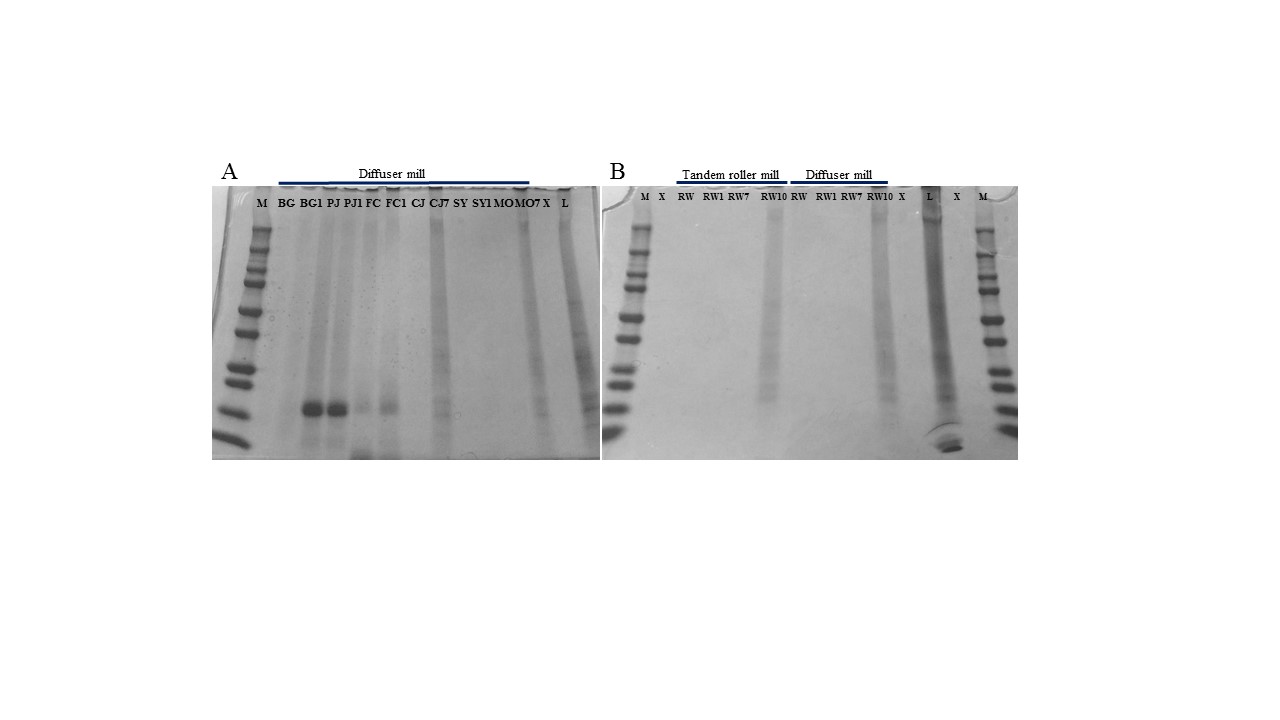

Supplement: Figure S1 — SDS-PAGE gel of protein in fractions of Brazilian mills. M, molecular 838 Marker; BG, bagasse; PJ, primary Juice; FC, Filter Cake; CJ, Clarified juice; SY, syrup; MO, 839 molasses; X, empty lane, L, leaf; RW, Raw sugar. Number following letters indicates spiking of 840 correspondent amount (in μg) of total total protein before protein extraction. [file image_1.jpeg]
