# Supplementary material for: Lack of Detection of Bt Sugarcane Cry1Ab and NptII DNA and Proteins in Sugarcane Processing Products Including Raw Sugar
Source: Front Bioeng Biotechnol. 2018 Mar 27;6:24. doi: 10.3389/fbioe.2018.00024 (PMC5880997; doi:10.3389/fbioe.2018.00024)
Supplement: Table S1 — Supplemental methodology. [file Table_1.docx]

**Supplemental Methodology – S1**

| **Filterability methodology** | Place 50.0 g +/- 0.5 g of sugar sample to be tested and dissolve it in 50.0 g +/- 0.5 g of deionized water; build the filter assembly with the filtering membrane; adjust the temperature of the solution to 25 °C +/- 1 °C; connect to the vacuum system in filter assembly, set to 15 inch vacuum +/- 1 inch mercury; transfer the sugar solution to the filter system and wait for one minute; start the vacuum and the timer and initiate the measurement of filtration time; If, after 5 minutes, the filtered volume is yet partial, close the test and measure the filtered volume in mL; the result is expressed in total volume filtered in minutes (mL - min). |
| --- | --- |
| **Acid Floc Methodology**  Modified from ICUMSA Method GS 2/3-40, 2007 | Place 55 g +/- 1g of sugar sample to be tested to 250 mL-Erlenmeyer and dissolve with 60 mL deionized water; connect to the vacuum system in filter assembly; filter the solution in pre-filter; collect the filtrate and transfer to 250 mL beaker; add 5 mL of 0.1% sodium benzoate solution (w / v), 4 mL of 0.67 mol / L of phosphoric acid and homogenize; transfer the solution to a graduated 500 mL-beaker and make up to volume with carbonated water (sparkling mineral water); transfer the entire solution into a 500 ml clear pet bottle and cap. Observe, with the help of light source, formation of flocs on the third, seventh and tenth day, avoiding sudden movements with the bottle to avoid breaking any flocs formed and assign a numerical index to each check. Assign after each observation a numerical index from 0 to 3 and express the result by the observed on the tenth day, as follows: 0 = Negative: no visible flakes / 1 = Light: Rare and light flakes; / 2 = Moderate: Light flocculation, easily visible with the aid of light source; / 3 = Heavy: Flakes observed without light source. |
| **Alcoholic Floc Methodology** | Place 50.0 g +/- 0.5 g of the sugar sample to be tested and dissolve it in 50.0 g +/- 0.5 g of deionized water; heat the solution to boiling point and filter in 0.8 μm membrane filtration; cool the filtered solution immediately to room temperature; place 49 mL of the filtered solution in a 250 mL Erlenmeyer; add 55 mL of deionized water and 120 mL of absolute ethyl alcohol p.a.; homogenize the solution, cap the vial with plastic film or cap; leave to stand for 15 min; adjust the spectrophotometer to a wavelength of 420 nm, use the 55% v / v alcohol solution as blank and make the absorbance readings in the cell of 10 mm; express the results in units of absorbance at 420 nm. |
